# Comprehensive Selection of Reference Genes for Gene Expression Normalization in Sugarcane by Real Time Quantitative RT-PCR

**DOI:** 10.1371/journal.pone.0097469

**Published:** 2014-05-13

**Authors:** Hui Ling, Qibin Wu, Jinlong Guo, Liping Xu, Youxiong Que

**Affiliations:** Key Laboratory of Sugarcane Biology and Genetic Breeding, Ministry of Agriculture, Fujian Agriculture and Forestry University, Fuzhou, Fujian, China; Zhejiang University, China

## Abstract

The increasingly used real time quantitative reverse transcription-PCR (qRT-PCR) method for gene expression analysis requires one or several reference gene(s) acting as normalization factor(s). In order to facilitate gene expression studies in sugarcane (*Saccharum officinarum*), a non-model plant with limited genome information, the stability of 13 candidate reference genes was evaluated. The geNorm, NormFinder and deltaCt methods were used for selecting stably expressed internal controls across different tissues and under various experimental treatments. These results revealed that, among these 13 candidate reference genes, *GAPDH*, *eEF-1a* and *eIF-4α* were the most stable and suitable for use as normalization factors across all various experimental samples. In addition, *APRT* could be a candidate for examining the relationship between gene copy number and transcript levels in sugarcane tissue samples. According to the results evaluated by geNorm, combining *CUL* and *eEF-1α* in hormone treatment experiments; *CAC* and *CUL* in abiotic stress tests; *GAPDH*, *eEF-1a* and *CUL* in all treatment samples plus *CAC*, *CUL*, *APRT* and *TIPS-41* in cultivar tissues as groups for normalization would lead to more accurate and reliable expression quantification in sugarcane. This is the first systematic validation of reference genes for quantification of transcript expression profiles in sugarcane. This study should provide useful information for selecting reference genes for more accurate quantification of gene expression in sugarcane and other plant species.

## Introduction

Real time quantitative reverse transcription-PCR (qRT-PCR) is increasingly used in gene expression analysis owing to its simple, reproducible and high-throughput features. qRT-PCR provides a useful and rapid means of understanding gene expression in living organisms by measuring the expression of target genes across different samples. In addition, qRT-PCR is a low cost and widely accepted method in the tracking of gene expression levels in genetically modified organisms (GMO) as well as in molecular breeding and gene mining. When performing qRT-PCR analysis, several factors such as sample amount, RNA integrity, cDNA quality, as well as the tissues or cell activities, can affect the quantitative measurement of gene expression [1]. Thus, in order to obtain a reliable analysis of gene expression by qRT-PCR, one or several reference genes should serve as the internal control to normalize and monitor the expression variation between samples and reactions [2]–[4]. The expression of these reference genes should remain stable under various experimental treatments and/or at different stages of development and growth periods [2]–[4]. Specifically, a suitable reference gene for performing qRT-PCR analysis should: (i) have stable expression across all or most of the samples analyzed; (ii) have no association with any pseudogene, to avoid the amplification of non-functional gene family members; (iii) reflect variations in RNA quality and quantity, as well as cDNA synthesis and amplification; (iv) possess the stability of transcription that is suitable for the target gene; and (v) exhibit moderate expression levels (i.e. a threshold cycle Ct of 15 to 30) [5].

Housekeeping genes (HKGs) related to basal cell activities and cellular structure components have been historically used as internal controls in medical science [6] and later in plant science [7]. The most commonly used HKGs are 25S *rRNA*, *GAPDH* (glyceraldehyde-3-phosphate dehydrogenase), *ACT* (β or γ actin) and *TUB* (α or β tubulin). However, using a genome wide approach, hundreds of genes in *Arabidopsis thaliana* were shown to outperform traditional reference genes in terms of expression stability throughout development and under a range of environmental conditions [8]. Among them, genes encoding a protein phosphatase 2A subunit, a coatomer subunit and an ubiquitin-conjugating enzyme were identified as novel reference genes [8].

Recently, a range of new reference genes have been validated across a set of tissues and differently treated samples by systematic statistical algorithms termed geNorm [2], BestKeeper [9], NormFinder [1], the deltaCt method [10] and the RefFinder WEB-based software (http://www.leonxie.com/reference gene.php) [11]. These genes include *UBQ5* (Ubiquitin5), *eEF-1a* (Eukaryotic elongation factor 1-alpha) and *eIF-4α* (Eukaryotic initiation factor 4-alpha) in *Oryza sativa*; *CUL* (Cullin), *FPGS* (Folylpolyglutamate synthase), *LUG* (Leunig), *MEP* (Membrane protein) and *UBCP* (Ubiquitin carrier protein) in *Zea mays*; *CAC* (Clathrin adaptor complex) and *TIPS-41* (Tonoplastic intrinsic protein41) in *Brassica juncea* as well as *APRT* (adenine phosphoribosyl transferase) in *Solanum tuberosum* and *Setaria italica* L. [12]–[17]. Zhang et al. investigated ten candidate genes in five different monocot plants (*Brachypodium beauv*, *Hordeum vulgare*, *Sorghum bicolor*, *Triticum aestivum* and *Z. mays*) under infection with different viruses. They found that *GAPDH* performed well in *B. beauv* and that *EF1α* (designated *eEF-1a* in the present study) performed well in *T. aestivum* [18]. Additionally, a number of reference genes have been validated in plants such as *Solanum tuberosum* [19], *Glycine max* [12], [16], [20], *Solanum lycopersicum* L. [21], [22], *Vitis vinifera* [23], [24], *T. aestivum* [13], [25], *Brassica napus* [26], cereals (*T. aestivum*, *H. vulgare* and *Avena sativa* L.) [27], *Cucumis sativus* Linn. [5], *Nicotiana tabacum* [28] and *Phyllostachys edulis* [29]. Lastly, Caus et al. identified endogenous reference genes in the base genome of sugarcane (*Saccharum officinarum*) [30], and two of them, *APRT* and *PRR* (Pseudo response regulator), are used in the present study to test the stabilities of “low copy number genes” in transcripts.

Sugarcane (*S. officinarum×S. spontaneum*) is a widely grown sugar crop in the tropics and subtropics with increasing demand due to biofuel production and challenges with biomass production [31]. Identifying genes for sucrose accumulation and stress resistance, which requires a set of reference genes for gene expression normalization, could serve to increase sugarcane yield and sucrose content using both genetically modified strategies and molecular marker-assisted breeding. Sugarcane has limited available genomic information so only a few of genes, mainly *GAPDH* and 25S *rRNA*, have been verified as practicable reference genes [32], [33]. However, there is expression variation of *GAPDH* and 25S *rRNA* under various experimental conditions [2], [12], [34].

In the present study, the 13 genes 25S *rRNA*, *GAPDH*, *ACT* (β-actin), *TUB* (β-tubulin), *APRT*, *PRR*, 18S *rRNA*, *eEF-1a*, *eIF-4α*, *CAC*, *TIPS-41*, *CUL* and *UBQ* were selected as candidate reference genes for evaluation in sugarcane. This study utilized 57 sugarcane samples and aimed to reveal which reference genes should be used in experimental samples with different treatments or different tissues. A combination of reference genes was also introduced to evaluate their potential for more accurate and reliable qRT-PCR analysis of gene expression in sugarcane.

## Materials and Methods

### Ethics Statement

No specific permissions were required for these locations/activities. The field studies did not involve endangered or protected species and the specific location of this study is longitude: 119.23E, latitude: 26.08N.

### Plant Materials, Growth Conditions and Treatments

Five sugarcane cultivars, “ROC”20, “ROC”22, FN40, Liucheng03-182 and YC05-179, were used for tissue sample collection. Five tissue samples were harvested (leaf, leaf sheath, stem epidermis, stem pith and bud) from 7- to 8-month-old sugarcane grown in the field. For each cultivar, 6 plants from the same experimental plot were collected to provide 6 replicates. The leaf and leaf sheath samples were taken from the last fully expanded leaf (+1 leaf), while the stem samples (epidermis and pith) and the buds were harvested from the 6^th^ or 7^th^ stem internodes. All materials were cut into small pieces, wrapped in tinfoil, immediately snap-frozen in liquid nitrogen and then kept at −80°C until RNA isolation.

Four sugarcane cultivars, “ROC”20, FN40, Liucheng03-182 and YC05-179, were used for the stress experiments. The single bud node shoots that used for *in vitro* disease-free plantlets regeneration, were incubated in 50°C water with the fungicide carbendazim (100 mg·L^−1^; Tianjin, China) for 40 min. The shoots were then planted in autoclaved soil before harvest for meristem excision, callus induction, shoot differentiation and rooting [35]. The plantlets were then placed in distilled water and kept for ten days in a tissue culture room under a constant temperature of 25±1°C. Different sets of plants were then transferred into test tubes containing water solutions along with different treatments, including abscisic acid (100 µM, ABA), methyl jasmonate (25 µM, MeJA) and salicylic acid (5 mM, SA) for 6 h; hydrogen peroxide (500 mM, H_2_O_2_), sodium chloride (250 mM, NaCl) and polyethylene glycol (25% w/v, PEG) for 12 h or copper chloride (100 µM, CuCl_2_) and cadmium chloride (500 µM, CdCl_2_) for 24 h. Each set of samples comprised three seedlings as biological replicates for expression analyses. The plants without treatment (kept in distilled water) were harvested as control. All materials were wrapped up in tinfoil, immediately snap-frozen in liquid nitrogen, and then kept at −80°C until RNA isolation.

A total of 57 samples (56 plus the control), including 25 tissue samples and 31 treated samples (the H_2_O_2_ treated sample of YC05-179 was absent) exposed to various stress treatments involving four different cultivars, were employed in the experiments for evaluation of candidate reference genes in sugarcane.

### RNA Isolation, DNase Treatment and cDNA Synthesis

TRIZOL reagent (Invitrogen Co., Carlsbad, CA, USA) was used in the RNA isolation of 25 tissue samples from cultivars “ROC”20, FN40, Liucheng03-182 and YC05-179 following the manufacturer’s instructions. These RNA samples were treated with RNase-free DNaseI (Promega, Fitchburg, WI, USA) before used in reverse transcription. A total of 31 plantlet samples were used for RNA extraction by the RNAprep Pure Plant Kit (polysaccharides & Poluphenalics-rich) (Tiangen, Beijing, China). The quality of all RNA samples was analyzed by the Synergy H1 Microplate Reader Multi-Mode (Bio-Tek, Winooski, VT, USA) with a 260/280 ratio from 1.9 to 2.1 and a 260/230 ratio from 2.0 to 2.5. The integrity of the RNA samples was analyzed by agarose gel electrophoresis.

The first-strand cDNA was synthesized with a 10 µL reaction system according to the instructions of the TAKARA PrimeScrit RT Reagent Kit (Perfect for Real Time) (TaKaRa Biotechnology, Dalian, China). The quality and integrity of cDNA were determined in the same way as above. All the cDNA samples were diluted to 5 ng·µL^−1^ for the following qRT-PCR reaction and stored at −20°C until use.

### qRT-PCR and Data Analyses

An ABI 7500 Real Time PCR machine (Applied Biosystems, Foster City, CA, USA) and its default program (2 min at 50°C and 10 min at 95°C followed by 40 cycles at 94°C for 15 s, and at 60°C for 60 s.) were employed for qRT-PCR with a reaction mixture volume of 20 µL in an optical 96-well plate. 10 µL of SYBR Green Master Mix (Roche), 10 pM of each primer, 10 ng of final cDNA and 6.4 µL of RNase-free water were added to the reaction mixture. A control was also included in each plate with 2.0 µL of RNase-free water as a template. Three technical replicates were contained in each plate. Specificity verification of the PCR amplification dissociation and the PCR efficiency curves were determined for each candidate reference gene prior to the qRT-PCR evaluation of these genes in sugarcane.

### Statistical Analysis of Gene Expression and Comparison of Normalization Methods

The PCR amplification efficiencies of all gene assays were estimated based on the slope of the standard curve in conjunction with the following formula:
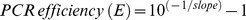
(1)


Standard, PCR efficiency and the correlation coefficient (R^2^) curves of each gene were generated in Microsoft Excel 2003 using a range of seven dilutions made in ten, five or three-fold decrements on the YC05-179 control sample. *GAPDH* and *TUB* were ten-fold; 25S *rRNA*, *ACT*, 18S *rRNA*, *eEF-1a*, *CUL* and *eIF-4α* were five-fold and *CAC*, *TIPS-41*, *UBQ*, *APRT* and *PRR* were three-fold.

The overall expression levels of the candidate genes were transformed into threshold cycle (Ct) values by the ABI 7500 Fast Real Time PCR System. Ct values over 40 indicated undetectable product and were considered as missing values (NA) for subsequent calculations. After collecting and converting the threshold PCR cycle data, Ct average values for the evaluated genes were inputted into software according to the corresponding manuals of geNorm (trial version; Biogazelle, Zwijnaarde, Belgium) [2] and NormFinder (ver. 0.953) [1]. After that, the values of stability of candidate genes achieved from geNorm, NormFinder and deltaCt (only the deltaCt results from RefFinder were employed [16]) were used to calculate Pearson correlation values (r value) by SAS S21.0. The values reflect the level of correlation of between the results from geNorm, NormFinder and deltaCt.

## Results

### Screening of Candidate Reference Genes and Primer Design

A total of 13 candidate reference genes were selected in sugarcane or in other plant species for evaluation on the basis of their stable expression across developmental stages and/or abiotic stresses. These included four previously assessed sugarcane candidate genes, 25S *rRNA*, *GAPDH*, *ACT* and *TUB* [31], [32], as well as nine new candidate reference genes, *APRT*, *PRR*, 18S *rRNA*, *eEF-1a*, *eIF-4α*, *UBQ*, *CAC*, *TIPS-41* and *CUL*. Since there is limited available sugarcane genome sequence information, the publicly available gene sequences from *O. sativa* (18S *rRNA*, AK059783; *eEF-1a*, AK061464; *eIF-4α*, AK073620; *UBQ5*, AK061988), *Z. mays* (*CUL*, GRMZM2G166694_T04) and *A. thaliana* (*CAC*, *TIPS-41*) were used as the probes to search within a sugarcane expressed sequence tags (ESTs) database (www.ncbi.nlm.nih.gov/nucest/?term=sugarcane). Two candidate reference genes in sugarcane which performed well in *B. juncea* [12], named *CAC* and *TIPS-41*, were identified by querying homologous sugarcane sequences with *A. thaliana* genes complete CDS (*CAC*, At5G46630; *TIPS-41*, At4G34270). The remaining five new candidates, 18S *rRNA*, *eEF-1a*, *eIF-4α*, *UBQ*, and *CUL*, performed well in *O. sativa* [9] and *Z. mays* [11]. All seven EST sequences, including those of 18S *rRNA*, *eEF-1a*, *eIF-4α*, *UBQ5*, *CAC*, *TIPS-41* and *CUL*, were acquired from the publicly available database in NCBI using candidate ESTs with the highest homology to the target sequences. Both reference sequences from *O. sativa*, *Z. mays*, *B. juncea*, *T. aestivum* or *A. thaliana* and the corresponding target sequences from sugarcane were aligned together in DNAMAN to identify the complete sequence identity. This information was used to design primers using the Primer-BLAST tool from the NCBI (http://www.ncbi.nlm.nih.gov/tools/primer-blast/). The primer sequences for all 13 candidate reference genes are shown in Table 1.

**Table 1:** Thirteen reference genes surveyed in this work with their amplification and expression characteristics in *Saccharum officinarum.*

gene	Accessionnumber	Functions	Primer F/R (5′–3′)	Ampliconlength (nt)	Tm (°C)	PCR efficiencies(E %)	RegressionCoefficient (R2)	MeanCt	SD	CV(%)	Reference
25S *rRNA*	BQ536525	25S ribosomal RNA	GCAGCCAAGCGTTCATAGC CCTATTGGTGGGTGAACAATCC	108	60	113.83	0.9982	14.27	0.71	5.01	*Saccharum*spp.L. [31]
*GAPDH*	CA254672	glyceraldehyde-3-phosphatedehydrogenase	CACGGCCACTGGAAGCA TCCTCA GGGTTCCTGATGCC	101	60	93.24	0.9986	24.64	1.23	5.01	*Saccharum*spp.L. [31]
*ACT* ^*^	CA148161	β-actin	CTGGAATGGTCAAGGCTGGT TCCTTCTGTCCCATCCCTACC	112	60	109.06	0.9988	25.03	1.06	4.23	*Saccharum*spp.L. [31]
*TUB* ^**^	CA222437	β-tubulin	CCAAGTTCTGGGAGGTGATCTG TTGTAGTAGACGTTGATGCGCTC	103	60	94.76	0.9997	26.91	1.43	5.31	*Saccharum*spp.L. [31]
18S *rRNA*	SCFRRE06	18S ribosomal RNA	CTACGTCCCTGCCCTTTGTACA ACACTTCACCGGACCATTCAA	65	60	97.24	0.9982	15.38	0.74	4.81	*Oryza sativa* [9]
*UBQ*	CA262530.1	Ubiquitin	ACCACTTCGACCGCCACTACTG CACCACCTAGCAA GGCTTTCCATTTC	69	60	102.3	0.9998	26.47	1.29	4.88	*Oryza sativa* [9]
*eEF-1a*	EF581011.1	Eukaryotic elongationfactor 1a	TTTCACACTTGGAGTGAAGCAGATGACTTCCTTCACAA TCTCATCATAA	103	60	96.87	0.9998	24.24	1.33	5.48	*Oryza sativa* [9]
*eIF-4α*	CA275432.1	Eukaryotic initiationfactor 4a	TTGTGCTGGATGAAG CTGATG GGAAGAAGC TGGAAGATATCATAGA	76	60	98.87	0.9876	27.98	1.24	4.42	*Oryza sativa* [9]
*CUL*	CF574093.1	Cullin	TGCTGAATGTGTTG AGCAGC TTGTCGCGCT CCAAGTAGTC	105	60	105.66	0.999	27.05	1.49	5.52	*Zea mays* [11]
*CAC*	CA203604.1	Clathrin adaptor complex	ACAACGTCAGGCAAAGCAAA AGATCAACTCCACCTCTGCG	112	60	99.5	0.9999	27.88	1.41	5.05	*Brassica juncea* [12]
*TIPS-41*	CA228782.1	Tonoplastic intrinsic protein	CACCTGTTGAGGTTCCTGCT CACAGCATCACTCCCACAGT	116	60	113.56	0.9964	28.09	2.15	7.67	*Brassica juncea* [12]
*APRT*	CA089592.1	Anthranilate phosphoribosyltransferase	TGACACATTCCCAACCTCAA ATCTCTCTCCCTGAGTGGCA	119	60	102.53	0.9993	27.35	1.37	5	*Saccharum* spp.L.[29]
*PRR*	CA275446.1	Pseudo response regulator	GCCAAATTCAGGCAGAAAAG CACCCTAGGCCTTGTTTCAG	93	60	98.13	0.9999	28.21	2.04	7.25	*Saccharum* spp.L.[29]

Mean Ct values and RNA concentration were used for calculating slopes and correlation coefficients (R^2^). According to the formula [E = (10^(−1/slope)^−1)×100%, qPCR efficiencies (E) were calculated based on the standard curves. Mean Ct value (mean), Standard deviation (SD) and Covariance (CV) were calculated by Microsoft Excel 2003 and the Ct values from all of the samples were used. The sequence numbers were obtained from www.ncbi.nlm.nih.gov/nucest/?term=sugarcane.

### Verification of Primer Specificity, Efficiency and Gene Expression Profile

Using the control sample of YC05-179 as the cDNA template, the specificity of primers used in qRT-PCR experiments was confirmed based on a melting curve analysis and agarose gel electrophoresis. The standard curve, PCR efficiency and the correlation coefficient (R^2^) of each gene were generated in Microsoft Excel 2003 using a range of seven dilutions made in ten (*GAPDH* and *TUB*), five (25S *rRNA*, 18S *rRNA*, *eEF-1a*, *eIF-4α* and *CUL*) or three-fold (*CAC*, *TIPS-41*, *UBQ*, *APRT* and *PRR*) decrements on the YC05-179 control sample. The qRT-PCR efficiency formula (Eq. 1) was used in the calculation. The regression coefficient R^2^ for all the primers varied between 0.9876–0.9999 over a serial dilution of cDNA. qRT-PCR efficiencies of primers ranged from 93.24% to 113.83% (Table 1).

Over all samples, the Ct values of the 13 candidate reference genes varied over a wide range, and the mean Ct values of these genes across all the samples ranged from 14.27 to 28.21 (Table 1). Among these candidate reference genes, 25S *rRNA* was the most abundantly expressed gene in all of the samples (mean Ct±SD = 14.27±0.71) followed by 18S *rRNA* (mean Ct±SD = 15.38±0.74), whereas *PRR* was the least abundantly expressed gene (mean Ct = 28.21±2.04). *ACT*, *GAPDH* and *eEF-1a* were close in Ct values (25.03, 24.64 and 24.24, respectively) but differed in SD values (1.06, 1.23 and 1.33, respectively). The mean Ct±SD of the remaining genes (*TUB*, *TIPS-41*, *CAC*, *CUL*, *eIF-4α*, *UBQ*, *APRT* and *PRR*) varied from 26.47±1.29 to 28.21±2.04. The data also showed that the expression of *ACT* and *eIF-4α* were the least variable (Covariance (CV) of 4.23% and 4.42%, respectively). The CV values of 18S *rRNA*, *UBQ*, 25S *rRNA*, *GAPDH*, *CAC* and *APRT* ranged from 4.81% to 5.05%. The CV values of *TUB*, *eEF-1a* and *CUL* ranged from 5.31% to 5.52%. In contrast, the CV values of *TIPS-41* and *PRR* were 7.67% and 7.25%, respectively (Table 1).

In order to make a wider expression analysis of candidate reference genes, the 57 diverse samples were divided into four experimental sets. The 1^st^ set consisted of leaf, leaf sheath, stem epidermis, stem pith and bud samples from “ROC”20, “ROC”22, FN40, Liucheng03-182 and YC05-179 (Table 2). The 2^nd^ set was comprised of 12 samples from FN40, Liucheng03-182, “ROC”20 and YC05-179 treated with ABA, MeJA and SA. The 3^rd^ set contained 19 samples treated with H_2_O_2_, NaCl, PEG, CuCl_2_ and CdCl_2_ (abiotic stresses). In the 4^th^ set, both the 2^nd^ and 3^rd^ sample sets were included. The variation of transcript levels indicated that the expression of the candidate genes was affected by tissue types and experimental conditions (Table 2). In the 1^st^ set, 18S *rRNA* performed as the least variable gene, whereas 25S *rRNA*, *CUL* and *PRR* had a larger range of expression level variation. 25S *rRNA* and *eIF-4α* had the lowest expression variation in the 2^nd^ set and, 25S *rRNA* and *ACT* had the lowest variation in the 4^th^ set. *ACT* and *TUB* performed less variability in the 3^rd^ set. Additionally, *TIPS-41&TUB* and *TIPS-41&PRR* displayed the most variable expression profiles when treated with hormones and abiotic stresses, respectively (Table 2). A significant variation in the expression of *PRR* and *APRT* was observed in all treatment samples (Table 2). Although none of the candidate reference genes displayed constant expression levels throughout the various cultivars and among the different kinds of treatments, six genes among these 13 reference genes (25S *rRNA*, 18S *rRNA*, *GAPDH*, *ACT*, *UBQ* and *eIF-4α*) varied at a relatively lower level according to CV values (Table 2).

**Table 2:** Expression levels and variation of 13 reference genes across four experimental sets in *Saccharum officinarum.*

Set		25S *rRNA*	18S *rRNA*	*GAPDH*	*ACT*	*TUB*	*TIPS-41*	*CAC*	*CUL*	*UBQ*	*eEF-1a*	*eIF-4α*	*APRT*	*PRR*
	Mean	14.55	15.56	25.04	25.7	26.97	29.1	27.71	26.59	26.24	24.14	27.91	27.32	27.96
1st set	SD	0.83	0.4	0.79	0.91	1.04	1.09	1.33	1.37	1.3	0.96	0.98	1.14	1.43
	CV	5.72%	2.57%	3.16%	3.53%	3.86%	3.76%	4.78%	5.15%	4.94%	3.96%	3.52%	4.19%	5.10%
	Mean	13.88	15.28	23.95	24.46	27.22	27.5	27.53	26.88	26.29	23.81	27.72	26.59	27.15
2nd set	SD	0.27	0.95	0.74	0.79	2.47	2.41	0.92	0.94	0.74	0.88	0.75	0.99	1.07
	CV	1.96%	6.24%	3.08%	3.22%	9.07%	8.77%	3.35%	3.50%	2.82%	3.70%	2.69%	3.71%	3.94%
	Mean	14.28	15.39	24.71	24.9	26.89	27.9	28.26	27.59	26.81	24.66	28.35	27.82	29.05
3rd set	SD	0.74	0.83	1.63	0.97	0.91	2.22	1.67	1.72	1.52	1.85	1.75	1.67	2.88
	CV	5.15%	5.40%	6.59%	3.88%	3.39%	7.96%	5.91%	6.24%	5.68%	7.49%	6.16%	6.00%	9.90%
	Mean	14.08	15.25	24.38	24.58	26.87	27.42	27.99	27.35	26.62	24.31	28.02	27.37	28.37
4th set	SD	0.57	0.87	1.39	0.92	1.64	2.4	1.46	1.51	1.28	1.52	1.38	1.5	2.35
	CV	4.04%	5.72%	5.72%	3.73%	6.10%	8.75%	5.23%	5.51%	4.82%	6.27%	4.94%	5.50%	8.29%

Mean Ct value (Mean), Standard deviation (SD) and Covariance (CV) were calculated by Microsoft Excel 2003. 1^st^ set: leaf, leaf sheath, stem epidermis,stem pith and bud from “ROC”20, “ROC”22, FN40, liucheng03-182 and YC05-179; 2^nd^ set: ABA, MeJA and SA, treated-samples from “ROC”20, FN40, Liucheng03-182 and YC05-179; 3^rd^ set: H_2_O_2_, NaCl, PEG, CuCl_2_ and CdCl_2_ treated-samples from “ROC”20, FN40, liucheng03-182 and YC05-179; 4^th^ set: 2^n^set+3^rd^ set.

### Expression Stability Analysis and Ranking of All 13 Candidate Reference Genes in Sugarcane

**Figure 1:**
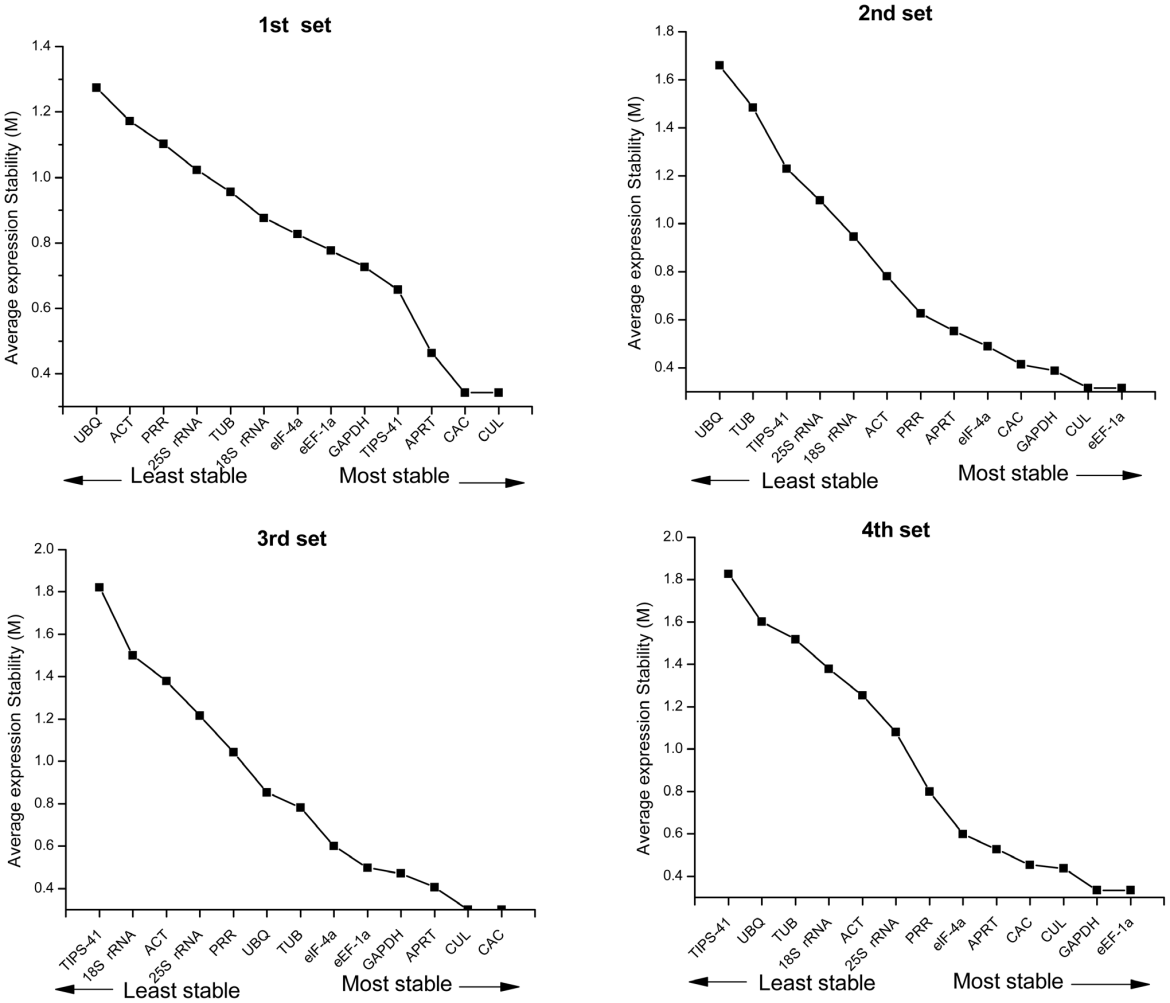
Gene expression stability of 13 candidate genes in sugarcane as predicted by geNorm. Average expression stability (M) following stepwise exclusion of the least stable gene across all the samples within an experimental set. The least stable gene is on the left, and the most stable on the right. The name *eIF-4a* in the figure stands for *eIF-4α. ACT* stands for “β-actin” and *TUB* stands for “β-tubulin”.

Based on the expression stability analysis of the 13 candidate reference genes by geNorm, the six top ranked genes in different cultivar tissues were *CUL* = *CAC*>*APRT*>*TIPS-41*>*GAPDH*> *eEF-1a*. The top ranked genes in the cultured plantlets under hormone treatments were *CUL* = *eEF-1a*>*GAPDH*>*CAC*, and the rank of the top six genes in the 3d set was *CAC* = *CUL*> *APRT*>*GAPDH*>*eEF-1a* (Fig. 1). The top six genes that were expressed relatively constantly in the 4^th^ set were *eEF-1a* = *GAPDH*>*CUL*>*CAC*>*APRT*>*eIF-4α* (Fig. 1). It is interesting to note that the most stable traditional housekeeping gene was *GAPDH* compared to 25S *rRNA*, *ACT* and *TUB*, which is similar to previous reports [32], [33]. Three reference genes, the *eEF-1a*, *CAC* and *CUL,* were more stable than the other six new candidates (18S *rRNA*, *eIF-4α*, *UBQ*, *TIPS-41*, *APRT* and *PRR*). Also, in response to various abiotic stresses and hormone treatments, *UBQ* or *TIPS-41* were identified as the least stable genes (Fig. 1).

NormFinder analysis revealed that *eEF-1a* and *eIF-4α* were always two of the top three most stable reference genes in the treated samples, while the third place was occupied by *ACT*, *GAPDH* or *TUB* in the 2^nd^, 3^rd^ and 4^th^ sets, respectively (Table 3). Three reference genes, *GAPDH*, *eEF-1a* and 18S *rRNA*, were most stable in the different tissues from all the five tested cultivars (Table 3). *UBQ* had the worst stability of the 13 candidate reference genes in untreated samples and in samples under hormone treatments (Table 3). Likewise, *TIPS-41* ranked at the bottom of these candidate genes in both the 3^rd^ and 4^th^ sets (Table 3). Though *ACT*, *TUB* and *GAPDH* expressed at less variable levels in the 1^st^, 2^nd^ and 4^th^ sets, the expression of all the four traditional housekeeping genes fluctuated across all four experimental sets and was less stable than *eIF-4α* or *eEF-1a* in most of the samples (Table 3).

**Table 3:** Expression stability of 13 reference genes in *Saccharum officinarum* as calculated by Normfinder.

Rank	1^st^ set		2^nd^ set		3^rd^ set		4^th^ set	
	gene	stability	gene	stability	gene	stability	gene	stability
1	*GAPDH*	0.053	*eIF-4α*	0.288	*eIF-4α*	0.269	*eIF-4α*	0.218
2	*eEF-1a*	0.148	*ACT* *	0.385	*eEF-1a*	0.462	*eEF-1a*	0.419
3	18S *rRNA*	0.156	*eEF-1a*	0.466	*GAPDH*	0.506	*TUB* **	0.419
4	*eIF-4α*	0.167	*CUL*	0.557	*TUB* **	0.583	*UBQ*	0.445
5	*APRT*	0.253	*PRR*	0.573	*UBQ*	0.620	*GAPDH*	0.505
6	25S *rRNA*	0.255	*GAPDH*	0.583	*CUL*	0.666	*ACT* *	0.546
7	*CAC*	0.256	*APRT*	0.615	*APRT*	0.671	*CUL*	0.571
8	*TIPS-41*	0.258	18S *rRNA*	0.656	*ACT* *	0.680	*APRT*	0.580
9	*CUL*	0.302	*CAC*	0.738	*CAC*	0.701	*PRR*	0.616
10	*TUB* **	0.315	25S *rRNA*	0.816	*PRR*	0.729	*CAC*	0.668
11	*ACT* *	0.335	*TIPS-41*	0.931	25S *rRNA*	0.748	25S *rRNA*	0.744
12	*PRR*	0.417	*TUB* **	0.961	18S *rRNA*	0.918	18S *rRNA*	0.787
13	*UBQ*	0.494	*UBQ*	0.990	*TIPS-41*	1.352	*TIPS-41*	1.092

*β-actin,

**β-tubulin.

The deltaCt method was used in RefFinder, which integrates geNorm, NormFinder, BestKeeper and deltaCt into a web-based program. The ranking of the 13 genes based on the deltaCt was consistent with that of NormFinder in different tissue samples except for the ranking of 25S *rRNA* (Table 3 and Table 4). Comparing the average expression stability of the top six genes valued by geNorm, NormFinder and deltaCt, the three candidate genes *GAPDH*, *eEF-1a* and *eIF-4α* performed better in all the 31 treated samples, and *eEF-1a*, *APRT* and *GAPDH* performed better in all the 25 tissue samples (Table 4). Conversely, using the three algorithms, *TIPS-41* and *UBQ* were the worst performing genes of all treated samples and untreated samples, respectively (Table 4). Pearson correlations were calculated among the geNorm, NormFinder and deltaCt methods using the stability values in the 1^st^ and 4^th^ sets. Interestingly, the Pearson correlations among all three stability tests were positive. A significant correlation was observed between outcomes from NormFinder and deltaCt (r = 0.946), indicating that the ranking results of all 13 reference genes from the above two methods were nearly identical in the 1^st^ set (Table 5). The lower correlations (Table 5) between geNorm and NormFinder in the 4^th^ set (r = 0.438) and the 1^st^ set (r = 0.476) were reflected by the results of the different rankings (Table 4).

**Table 4:** Comparison of the expression stability of 13 reference genes in *Saccharum officinarum* as calculated by geNorm, Normfinder and deltaCt.

Rank	1^st^ set						4^th^ set					
	geNorm		NormFinder		deltaCt		geNorm		NormFinder		deltaCt	
	gene	stability	gene	stability	gene	stability	gene	stability	gene	stability	gene	stability
1	*CAC*	0.343	*GAPDH*	0.053	*GAPDH*	0.940	*GAPDH*	0.333	*eIF-4α*	0.218	*CAC*	1.060
2	*CUL*	0.343	*eEF-1a*	0.148	*eEF-1a*	1.040	*eEF-1a*	0.333	*eEF-1a*	0.419	*eIF-4α*	1.070
3	*APRT*	0.464	18S *rRNA*	0.156	*1*8S *rRNA*	1.070	*CUL*	0.438	*TUB* **	0.419	*GAPDH*	1.080
4	*TIPS-41*	0.657	*eIF-4α*	0.167	*eIF-4α*	1.100	*CAC*	0.453	*UBQ*	0.445	*CUL*	1.090
5	*GAPDH*	0.726	*APRT*	0.253	*TIPS-41*	1.130	*APRT*	0.526	*GAPDH*	0.505	*eEF-1a*	1.100
6	*eEF-1a*	0.777	25S *rRNA*	0.255	*APRT*	1.130	*eIF-4α*	0.598	*ACT* *	0.546	*UBQ*	1.110
7	*eIF-4α*	0.827	*CAC*	0.256	*CAC*	1.190	*PRR*	0.800	*CUL*	0.571	*APRT*	1.160
8	18S *rRNA*	0.876	*TIPS-41*	0.258	*CUL*	1.220	25S *rRNA*	1.080	*APRT*	0.580	25S *rRNA*	1.220
9	*TUB* **	0.955	*CUL*	0.302	25S *rRNA*	1.300	*ACT* *	1.254	*PRR*	0.616	18S *rRNA*	1.390
10	25S *rRNA*	1.023	*TUB* **	0.315	*TUB* **	1.300	18S *rRNA*	1.378	*CAC*	0.668	*ACT* *	1.480
11	*PRR*	1.102	*ACT* *	0.335	*ACT* *	1.430	*TUB* **	1.518	25S *rRNA*	0.744	*PRR*	1.930
12	*ACT* *	1.172	*PRR*	0.417	*PRR*	1.560	*UBQ*	1.602	18S *rRNA*	0.787	*TUB* **	2.190
13	*UBQ*	1.274	*UBQ*	0.494	*UBQ*	1.810	*TIPS-41*	1.827	*TIPS-41*	1.092	*TIPS-41*	2.750

*β-actin,

**β-tubulin.

**Table 5:** Correlation coefficients based on the visualizing reference genes ranked by geNorm, Normfinder and deltaCt.

	1^st^ set	4^th^ set
geNorm *VS* NormFinder	0.476**	0.438**
geNorm *VS* deltaCt	0.679**	0.674**
NormFinder *VS* deltaCt	0.946***	0.549**

The p-value indicated by asterisks (***<0.0001; **<0.05).

### Optimal Number of Reference Genes for Expression Normalization across Different Experimental Sets

**Figure 2:**
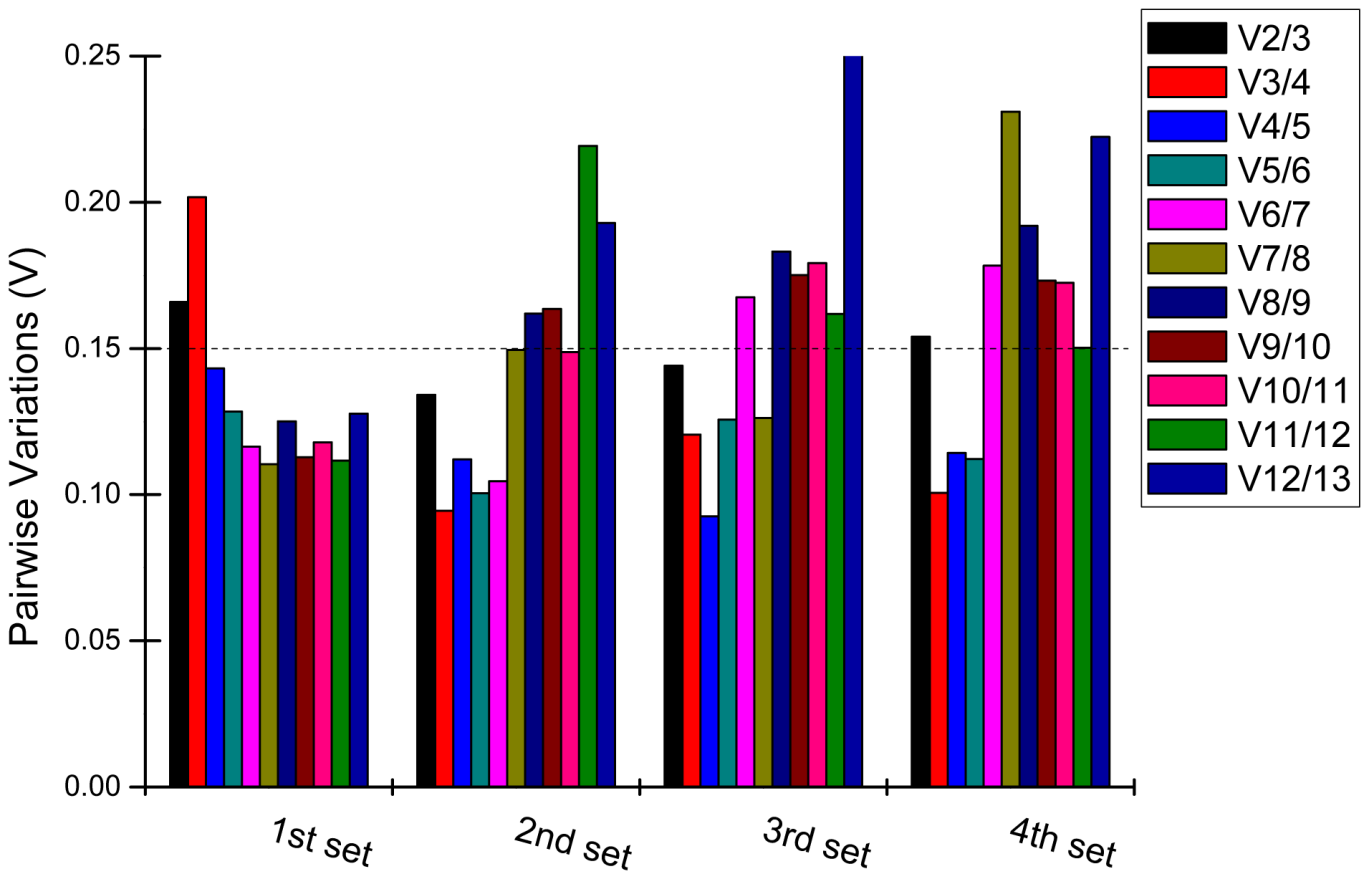
The optimal number of reference genes required for effective normalization in each of four experimental sets in sugarcane. The pairwise variation (V_n_/V_n+1_) was analyzed between normalization factors NF_n_ and NF_n+1_ by geNorm program to determined the optimal number of reference genes for accurate normalization in samples from different sugarcane cultivar samples (1^st^ set), sugarcane hormone-treated (2^nd^ set), abiotic-treated (3^rd^ set) and treatments (hormone-& abiotic-treated, 4^th^). *ACT* stand for “β-actin” and *TUB* stand for “β-tubulin”.

The optimal number of reference genes in the normalizing experiment was determined by geNorm by calculating the pairwise variation (V_n_/V_n+1_) between the normalization factors (NF) across all the samples of the different experimental sets. The pairwise variation (V = V_n_/V_n+1_), which was counted between NF_n_ and NF_n+1_, was used to find the best combination of genes for reliable normalization [2]. Vandesompele et al. suggested a threshold of V = 0.15, which indicates that adding one more gene has little influence on the calculation of normalization factor [2]. As shown in Figure 2, the rank order of gene stability established by means of stepwise exclusion of the least stable gene suggested that the combination of *CAC*, *CUL*, *APRT* and *TIPS-41* could provide a dependable result while normalizing the qRT-PCR data of the target gene in the 1^st^ sample set (Fig. 2). However, in the 2^nd^ set the two reference genes (*CUL* and *eEF-1a*) were enough to achieve a V_2/3_ = 0.134, which is close to the V = 0.15 threshold proposed by Vandeompele et al [2] and is thus an efficient and economical strategy to quantify sugarcane samples from hormone treatments. Similarly, the use of *CAC* and *CUL* was enough to achieve a V_2/3_ = 0.144 in samples treated with compounds eliciting abiotic stress (Fig. 2). The V_3/4_ value in the 4^th^ set samples from four sugarcane cultivars was 0.101 which suggests that the combination of *GAPDH*, *eEF-1a* and *CUL* is the best choice for quantification of gene expression in qRT-PCR (Fig. 2).

## Discussion

Sugarcane, sorghum, maize and rice belong to the Andropogoneae tribe and share many similarities in their genetic composition [36]–[40]. Rice, *O. sativa*, which diverged from a common Andropogoneae ancestor around 50–70 million years ago, performs as a model plant for grass species in several fields of modern molecular biology research [41]. Modern sugarcane cultivars are hybridized crosses between *S. officinarum* and *S. spontaneum*, resulting in a high degree of polyploidy and frequent aneuploidy [42]. Comparisons involving sugarcane as well as *O*. *sativa*, *S. bicolor* and *Z. mays* indicated significant common conservation of gene content and a few rearrangements [40]. The results of the present study also showed that the stable expression of candidate genes, such as *eIF-4α*, may follow the same pattern in *S. officinarum*, *O*. *sativa*, *S. bicolor* and *Z. mays*.

qRT-PCR is a quick, reliable and accurate tool to analyze gene expression. Accurate mRNA normalization requires one or two internal control genes which are stably expressed should be used. Since no single control is appropriate for all experimental treatments, it is generally suggested to select suitable internal controls prior to use for normalization. Although it is generally considered that housekeeping genes are expressed constantly, Lilly et al. and Nicot et al. revealed that the expression of such genes can undergo significant stability changes during biotic or abiotic stresses [18], [43]. Thus, several statistical algorithms, such as geNorm [2], NormFinder [1], BestKeeper [17], the deltaCt method [10] and the RefFinder WEB-based software [11], were developed to assess expression stability. Among these algorithms, geNorm, NormFinder and the deltaCt method were used in the present work. The deltaCt results were obtained from RefFinder, a web-based tool that integrates the most commonly used statistical algorithms. The normalization results were mostly affected by variation of the quantity and quality of introducing RNA and cDNA or even reaction-to-reaction variations [1].

When employing a variable reference gene, Gutierrez et al. revealed that nearly 100-fold variations could be found when quantifying target gene expression, thus leading to a huge potential scope for misinterpretation of the expression pattern of target genes [7]. Guo et al. observed much different expression values during a study of sugarcane dirigent protein gene expressed in sugarcane stems when using the different internal control genes of 25S *rRNA* and *GAPDH* [44]. Therefore, to achieve a reliable result with qRT-PCR, a systematically validated reference gene should be used and taken as an essential component of qRT-PCR analysis. In the present study, the three statistical algorithms, geNorm, Normfinder and deltaCt, were used and the Ct values were inputted into RefFinder. When correlation analysis based on the ranking order of the 13 candidate genes was applied for comparison of the three statistical algorithms, the results showed that the correlation coefficient between NormFinder and deltaCt was more positive (significantly in the cultivar set, r = 0.946) than the correlations between geNorm and Normfinder or between geNorm and deltaCt. These results were in accordance with the results of Jacob et al. [45]. The ranking order of the evaluated candidate genes by NormFinder and deltaCt in our study was generally consistent.

According to the CV values, 25S *rRNA*, 18S *rRNA*, *GAPDH*, *ACT*, *CAC, eIF-4α*, *UBQ* and *APRT* showed low variation in gene expression in all four experimental sets (Table 2). However, only *GAPDH*, *eEF-1a* and *eIF-4α* performed well in all three statistical algorithms. These three genes had relatively stable expression in different sugarcane genotypes and tissues. Our results also showed that the three genes *CUL*, *eEF-1a* and *eIF-4a* were suited for gene expression normalization in hormone-treated experiments, while *GAPDH* and *eEF-1a* were ideal when analyzing samples comparable to the 3^rd^ sample set. However, when these two sets (the 2^nd^ and 3^rd^ sets) were integrated into the 4^th^ set and analyzed together, *eEF-1a*, *eIF-4α* and *GAPDH* were the most stable reference genes.

In previous studies, *GAPDH* was found to possess the most stable mRNA expression in sugarcane [32] and 25S *rRNA* was the most stable gene in sugarcane infected with *Ustilago scitaminea* [33]. Similarly, this study identified *GAPDH* as one of the most stable reference genes for all types of hormone and abiotic treatments. 25S *rRNA* and 18S *rRNA* were two of the more abundantly expressed target genes under investigation, which is contrary to the reference gene selection principle that emphasizes moderate expression levels. It is difficult to detect the variation of 25S *rRNA* and 18S *rRNA* due to their rich content, so these two genes may not be ideal for expression normalization. It should be pointed out that some genes with stable but low expression levels, such as genes coding for certain transcription factors may be used as internal references in qRT-PCR experiments. A suitable reference should have a minimum difference in Ct value since this would lessen influence on the quantification and hence be more accurate [8], [54]. In the present study, four historically used housekeeping genes, *GAPDH*, 25S *rRNA*, *ACT* and *TUB*, were also included in the evaluation [32], [33]. 25S *rRNA*, *ACT* and *TUB* had poor performance across all four experimental sets of sugarcane samples, and similar poor performance was seen when using some of the novel candidates, such as *UBQ*, *TIPS-41*, *PRR* and 18S *rRNA*.

In this study, *eEF-1a*, which has been proven to be a suitable reference gene for expression normalization in *O. sativa* and *C. sativus* [5], [12], also ranked at the top when evaluated by geNorm, NormFinder and deltaCt across our four experimental sets. Being an important specific protein factor involved in the process of protein translation, *eIF-4α* had the same performance as *eEF-1a* in most experimental treatment conditions in our study. Our study showed that *eIF-4α* had a relatively high expression under most experimental conditions, which is in agreement with the study of Zhu et al. in *C. papaya* [46]. *eIF-4α* also performed well in *Musa paradisiaca*, *Lycoris longituba*, *Hevea brasiliensis* and *Coffea* spp. [47]–[50]. The low copy number of *APRT* in the sugarcane genome, which was reported by Casu et al. [30], provides an advantage in analyzing gene copy number. However, in the present study the expression of *APRT* was found to be easily affected by abiotic stress conditions (3^rd^ set) and thus its application is limited. With more copies in the sugarcane genome than *APRT* [30], *PRR* displayed variable performance under different stresses and in different sugarcane tissues.

In other plants such as *B. juncea* [15], *S. lycopersicum* [22], *P. edulis* [29], *C. papaya* [46], *G. max* [51] and *L. culinaris* [52], the expression profiles of housekeeping genes, such as *GAPDH*, *eIF-4α* and *eEF-1a* were not expressed as consistently as non-traditional housekeeping genes such as *CAC*, *UBQ9*, *TIPS-41*, *NTB*, *ELF1b/60s*, *TBP1*, *TBP2*, *TIF*, *RPL2* and *PP2Acs*. However, in the present study *GAPDH*, *eEF-1a* and *eIF-4a* showed good stability in expression in different tissues from different genotypes and in samples treated with abiotic stress or biotic stress. The findings that *eEF-1a, eIF-4a* and *GAPDH* are expressed stably in sugarcane are also consistent with previous reports in *O. sativa* [12], [34], *S. tuberosum* [18], *C. papaya* [46], *Musa* (bananas and plantains) [53] and *N. tabacum* [54]. We therefore suggest that *GAPDH*, *eEF-1a* and *eIF-4a* should be considered as the most suitable candidate reference genes in sugarcane.

## Conclusion

In the present study, 13 candidate reference genes were evaluated by three independent analysis approaches according to stability of transcript profiles across sugarcane samples. The aim was to select the most suitable reference genes for further gene expression quantification in different tissues and stress-treated samples in sugarcane. The stability analysis of gene expression by geNorm, NormFinder and deltaCt revealed that *GAPDH*, *eEF-1a* and *eIF-4α* are the most suitable for normalization controls across different samples. This is the first systematic validation of reference genes for quantification of transcript profiles in sugarcane. In our study, combining different reference genes is advocated for reliable normalization in different experimental samples. In addition, we also provide the procedure for identifying suitable reference genes by qRT-PCR in detail, which will be helpful in other plant species. Therefore, this study should provide useful information for selecting reference genes for accurate quantification of gene expression in sugarcane and other plant species.
