# Correction: Comprehensive Selection of Reference Genes for Gene Expression Normalization in Sugarcane by Real Time Quantitative RT-PCR

**DOI:** 10.1371/journal.pone.0118444

**Published:** 2015-03-27

**Authors:** The PLOS ONE Staff

In the Funding section, the grant number for the Research Funds for Distinguished Young Scientists in Fujian Provincial Department of Education grant is incorrect. Please refer to the correct Funding section here.

This work was funded by National Natural Science Foundation of China (31271782), Research Funds for Distinguished Young Scientists in Fujian Provincial Department of Education (JA13090) and Research Funds for Distinguished Young Scientists in Fujian Agriculture and Forestry University (xjq201202). The funders had no role in study design, data collection and analysis, decision to publish, or preparation of the manuscript.

Additionally, there is an error in the Reference section of Table 1. Please refer to the correct Table 1 here.

**Table 1:** Thirteen reference genes surveyed in this work with their amplification and expression characteristics in *Saccharum officinarum*.

**gene**	**Accession number**	**Functions**	**Primer F/R (5'-3')**	**Amplicon length (nt)**	**Tm (°C)**	**PCR efficiencies (E %)**	**RegressionCoefficient (R^2^)**	**Mean Ct**	**SD**	**CV (%)**	**Reference**
25S *rRNA*	BQ536525	25S ribosomal RNA	GCAGCCAAGCGTTCATAGC CCTATTGGTGGGTGAACAATCC	108	60	113.83	0.9982	14.27	0.71	5.01	*Saccharum* spp.L. [32]
GAPDH	CA254672	glyceraldehyde-3-phosphate dehydrogenase	CACGGCCACTGGAAGCA TCCTCAGGGTTCCTGATGCC	101	60	93.24	0.9986	24.64	1.23	5.01	*Saccharum* spp.L. [32]
*ACT* ^*^	CA148161	β-actin	CTGGAATGGTCAAGGCTGGT TCCTTCTGTCCCATCCCTACC	112	60	109.06	0.9988	25.03	1.06	4.23	*Saccharum* spp.L. [32]
*TUB* ^**^	CA222437	β-tubulin	CCAAGTTCTGGGAGGTGATCTG TTGTAGTAGACGTTGATGCGCTC	103	60	94.76	0.9997	26.91	1.43	5.31	*Saccharum* spp.L. [32]
18S *rRNA*	SCFRRE06	18S ribosomal RNA	CTACGTCCCTGCCCTTTGTACA ACACTTCACCGGACCATTCAA	65	60	97.24	0.9982	15.38	0.74	4.81	*Oryza sativa* [12]
UBQ	CA262530.1	Ubiquitin	ACCACTTCGACCGCCACTACTG CACCACCTAGCAAGGCTTTCCATTTC	69	60	102.3	0.9998	26.47	1.29	4.88	*Oryza sativa* [12]
eEF-1a	EF581011.1	Eukaryotic elongation factor 1a	TTTCACACTTGGAGTGAAGCAGAT GACTTCCTTCACAATCTCATCATAA	103	60	96.87	0.9998	24.24	1.33	5.48	*Oryza sativa* [12]
eIF-4α	CA275432.1	Eukaryotic initiation factor 4a	TTGTGCTGGATGAAGCTGATG GGAAGAAGCTGGAAGATATCATAGA	76	60	98.87	0.9876	27.98	1.24	4.42	*Oryza sativa* [12]
CUL	CF574093.1	Cullin	TGCTGAATGTGTTGAGCAGC TTGTCGCGCTCCAAGTAGTC	105	60	105.66	0.999	27.05	1.49	5.52	*Zea mays* [14]
CAC	CA203604.1	Clathrin adaptor complex	ACAACGTCAGGCAAAGCAAA AGATCAACTCCACCTCTGCG	112	60	99.5	0.9999	27.88	1.41	5.05	*Brassica juncea* [15]
TIPS-41	CA228782.1	Tonoplastic intrinsic protein	CACCTGTTGAGGTTCCTGCT CACAGCATCACTCCCACAGT	116	60	113.56	0.9964	28.09	2.15	7.67	*Brassica juncea* [15>]
APRT	CA089592.1	Anthranilate phosphoribosyl transferase	TGACACATTCCCAACCTCAA ATCTCTCTCCCTGAGTGGCA	119	60	102.53	0.9993	27.35	1.37	5	*Saccharum* spp.L. [30]
PRR	CA275446.1	Pseudo response regulator	GCCAAATTCAGGCAGAAAAG CACCCTAGGCCTTGTTTCAG	93	60	98.13	0.9999	28.21	2.04	7.25	*Saccharum* spp.L. [30]

Mean Ct values and RNA concentration were used for calculating slopes and correlation coefficients (R2). According to the formula [E = (10(−1/slope)−1)×100%, qPCR efficiencies (E) were calculated based on the standard curves. Mean Ct value (mean), Standard deviation (SD) and Covariance (CV) were calculated by Microsoft Excel 2003 and the Ct values from all of the samples were used. The sequence numbers were obtained from www.ncbi.nlm.nih.gov/nucest/?term=sugarcane.

## References

[1] Ling H, Wu Q, Guo J, Xu L, Que Y (2014) Comprehensive Selection of Reference Genes for Gene Expression Normalization in Sugarcane by Real Time Quantitative RT-PCR. PLoS ONE 9(5): e97469 doi: 10.1371/journal.pone.0097469 24823940 10.1371/journal.pone.0097469 PMC4019594

